# Towards a prescribing monitoring system for medication safety evaluation within electronic health records: a scoping review

**DOI:** 10.1186/s12911-025-03096-3

**Published:** 2025-07-02

**Authors:** Camilo Scherkl, Theresa Dierkes, Michael Metzner, David Czock, Hanna M. Seidling, Walter E. Haefeli, Andreas D. Meid

**Affiliations:** 1 Internal Medicine IX, Department of Clinical Pharmacology and Pharmacoepidemiology, Medical Faculty/Heidelberg University Hospital, Heidelberg, Germany; 2 Internal Medicine IX, Department of Clinical Pharmacology and Pharmacoepidemiology, Cooperation Unit Clinical Pharmacy, Medical Faculty/Heidelberg University Hospital, Heidelberg, Germany

**Keywords:** Real-world evidence (RWE), Electronic health records (EHR), Clinical decision support system, Prediction model, Medication safety, Adverse drug events (ADE), Adverse drug reactions (ADR)

## Abstract

**Background:**

Medical care can fail for various reasons: diseases can remain undetected and their severity misjudged, therapies can be incorrectly dosed or ineffective, and therapies can trigger new conditions or adverse drug reactions (ADR). To manage the complexity of changing patient circumstances, data-driven techniques play an increasingly important role in monitoring patient safety and treatment success. Therefore, clinical prediction models need to consider longitudinal factors (“Prescribing Monitoring”) to ensure clinically meaningful results and avoid misclassification in the dynamic health situation of the individual patient.

**Methods:**

We have conducted a scoping review (OSF registration: 10.17605/OSF.IO/P93TZ) on prediction models for ADR to collect potential use cases for Prescribing Monitoring. This review identified 2435 relevant studies in English that were published in MEDLINE or EMBASE. Two reviewers screened the records for inclusion, with a third reviewer making the final decision in the event of discrepancies. In order to derive recommendations on the way towards a Prescribing Monitoring system, the following elements were extracted and interpreted: the prediction models used, selection of candidate predictors, use of longitudinal factors, and model performance.

**Results:**

A total of 56 studies were included after the screening process. We identified the main areas of current research in ADR prediction, all covering clinically important outcomes. We identified Prescribing Monitoring use cases based on their potential to (i) make individual predictions considering specific patient characteristics, (ii) make longitudinal predictions in a near time frame, and (iii) make dynamic predictions by updating predictions with previous risk predictions and newly available data. As a further aside, we use hyperkalaemia as an example to discuss the framework for developing Prescribing Monitoring in an electronic health record (EHR).

**Conclusion:**

This scoping review provides an overview of the use of time-varying effects and longitudinal variables in current prediction model research. For application to clinical cases, prediction models should be developed, validated and implemented on this basis, so that time-dependent information can enable continuous monitoring of individual patients.

**Supplementary Information:**

The online version contains supplementary material available at 10.1186/s12911-025-03096-3.

## Introduction

Medical care can fail for various reasons: undetected conditions (underuse) and misjudged severity, incorrectly dosing (medication error), ineffective treatments (nonresponse), or adverse drug reactions (ADR). Routine monitoring of disease triggers (e.g., viral load), disease activity (e.g., inflammatory markers [1]), treatment interventions (e.g., antimicrobial drug exposure [2]), and ADR (e.g., QT prolongation [3]) is therefore critical to ensure patient safety and that therapy is successful, especially when irreversible outcomes (e.g., death) must be prevented. Careful and proactive monitoring of any therapy is therefore essential.

The necessity, frequency, and the timing of the monitoring are influenced by disease progression, comorbidities, therapeutic response, ADR risk profiles, and dynamic clinical changes such as newly diagnosed conditions or changes in co-medication. These factors often require prompt, individualised and ongoing adjustment of the monitoring plan. This challenge is conceptually similar to the use of early warning scores in healthcare, which aim to identify acutely deterioration patients using static thresholds. However, while early warning scores are reactive tools designed for short-term deterioration detection, they often lack a clear methodology to include longitudinal information and multiple measurements within a patient [4]. Therefore, to manage this complexity, data-driven techniques – especially Machine-learning methods in electronic health records (EHR) – are increasingly used to detect conditions [5], predict clinically important outcomes [6], identify risk factors for ADR [7], identify the propensity of ADR in general [8], or the likelihood of specific ADR [9].

However, in order to achieve clinically meaningful results and avoid misclassification, the fundamentally dynamic health situation of the individual patient must be considered, which may include factors such as changes in health status, cumulative drug doses, and changes in prescriptions. Therefore, we define “Prescribing Monitoring” as a process for model-based detection of potentially critical situations and for alerting and advising prescribers during electronic prescribing on how to avoid harm to patients and address current risks. To be accurate, these risks should be (i) estimated individually, consider specific patient characteristics [10], and (ii) predict outcomes longitudinally over time [11]. The prediction model should (iii) be dynamically updated using population data and regularly reassess the accumulating data of the individual patient [12].

We assessed the current status of research in the field of clinical prediction models for ADR and conducted a scoping review to identify critical clinical situations where a Prescribing Monitoring is most promising. This informed the development of a generic methodological template for implementing such frameworks, which we briefly demonstrate using the prediction of potassium levels as an example.

## Methods

Our scoping review to identify prediction models for ADR in EHR was conducted in accordance with the JBI methodology for scoping reviews [13] and the PRISMA-ScR (Preferred Reporting Items for Systematic reviews and Meta-Analyses extension for Scoping Reviews) checklist (Supplementary Table S1, Additional File 1) [14]. It was conducted in accordance with an a priori protocol registered at the Open Science Framework in June 16th 2023 (10.17605/OSF.IO/P93TZ) [15].

### Study objectives

To identify situations where a Prescribing Monitoring system could be applied, we reviewed prediction models for ADRs developed in the last decade. In this scoping review, we focused on predictive models rather than diagnostic models. The specific objectives are:

1. Which ADR are chosen for clinical prediction models?
2. Which statistical methods are used and how successful are they in predicting outcomes?

While our aim is to inform developers of a Prescribing Monitoring system, numerous other reviews on the prediction of ADR in EHR [16–18], which depend on a variety of causative drugs [18], are going into more detail on the challenges of model implementation, adherence to reporting standards, and best practice for model development.

### Eligibility criteria

To develop the scoping review and select suitable studies, eligibility criteria were defined: (i) studies published from January 2013 to May 2025; (ii) written in English or German; (iii) addressing prediction models with focus on drug side effects; (iv) for hospitalised patients; (v) in EHR, computerised physician order entry or electronic clinical decision support systems (CDSS); and (vi) containing information on the statistical methods and evaluation of the model.

### Search strategy

An initial limited search of MEDLINE and Google Scholar was conducted to identify relevant articles on the topic. The keywords contained in the titles and abstracts of relevant articles and the index terms used to describe the articles were then used to develop a full search strategy for MEDLINE and EMBASE. The search strategy, including all identified keywords and index terms, was customised for each database (Table S2, Additional File 1). We screened publications of the last decade as we were more interested in recently developed models. The database searches were run in MEDLINE and EMBASE on the 12th May, 2025. The reference list of all included sources was reviewed for additional studies. Studies in English and German were considered.

### Study selection and data charting

The subsequent screening process consisted of (1) the review of title and abstract and (2) the reading of the full text. At the first level, two reviewers (CS and TD) screened all identified publications for the presence of minimal inclusion criteria. If one or both reviewers gave a positive evaluation, the respective article was selected for full text analysis. This was again conducted by both examiners checking for inclusion and exclusion criteria (Table S3, Additional File 1). If there was no agreement, a third reviewer (ADM) had the final decision. The screening was conducted with https://www.rayyan.ai/ [19]. Two reviewers (CS and ADM) determined the variables to be extracted and jointly developed a form for data collection. CS extracted the data independently and continuously updated the data collection form in an iterative process. The key information extracted concerned the target population, the predicted outcome, the model evaluation, and the statistical methodology of the prediction model.

### Data synthesis and analysis

All the extracted information was summarised and categorised in one table containing an overview and frequencies in a narrative format. The statistical conventional (logistic/linear regression, cox proportional hazard models, scores) and machine learning methods, predictor selection methods, and use of longitudinal variables were summarised. From the extracted information, we then derive a strategy for the development of an inpatient Prescribing Monitoring system to predict specific ADRs. The timeline of all steps during the scoping review process can be found in the appendix Table S5.

## Results

### Narrative summary of the literature review

After the screening of 2435 relevant publications, 56 studies were eligible (Table 1, Figure S1). The categories after extraction of information are summarised in Table 1. All studies include demographic data and prescriptions, 96% (54/56) also consider laboratory data, 93% (52/56) clinical data, and 29% (16/56) administrative data for the development of the model. In 68% (38/56) of the studies, it was specified how candidate predictors were identified. Among them, candidate predictor selection was based on literature reviews (38%) (21/56), clinical knowledge (30%) (17/56), previous studies (16%) (9/56), and in some cases included all variables (9%) (5/56) available in the databases. The variable selection during model development was described in 96% (54/56) of the studies. In 39 studies the handling of missing data was described: most studies used a form of simple imputation (mean, median, or mode) [3, 20–32], used a missing indicator [20, 22–24, 33–36], removed variables (e.g., with missing data above a certain threshold) [37, 38], applied a complete case analysis [25, 39, 40], or multiple imputation [37, 41–47]. In 70% (39/56) of the studies, multivariable logistic regression was used as a method to predict an ADR. Eighteen studies [26, 28, 31, 32, 35, 36, 38, 39, 43, 44, 47–54] developed multiple models to compare and identify the best model. Only 27% (15/56) of the studies incorporated longitudinal information, which cover summary statistics such as mean, maximum, minimum, trends, or other descriptive measures such as change from previous values, slopes, cumulative exposure of drugs or scores. These longitudinal predictors cover biochemistry data (e.g., blood glucose, potassium levels, and serum creatinine) [20, 21, 23, 24, 26, 32, 43, 49, 51, 54–56], vital signs (e.g., heart rate, respiratory rate) [21, 51], and prescription characteristics (e.g., coadministrations, cumulative doses, duration) [24, 39, 43, 46, 54].

**Table 1:** Description of the predicted adverse drug reactions, modelling methods, best performing method, best performance, and predictor selection in studies developing clinical prediction models for adverse drug reactions

Year	ADR	Method(s)	Best method	Best performance (AUC-ROC)	Longitudinal variables	Candidate predictor selection	Predictor selection during modelling	Reference
2018	Acute kidney injury	GBM	GBM	0.90	Discrete time survival analysis framework, min., max., slope, change	Not specified	Not specified	[21]
2019	Acute kidney injury	MLR	MLR	0.81	Trend, cumulative exposure	Literature review, clinical knowledge	UA, CA	[24]
2019	Acute kidney injury	CBI	CBI	0.76	No	Clinical knowledge, previous studies	Combinational interference	[57]
2019	Acute kidney injury	RNN	RNN	0.92	Count, mean, median, change	Not specified	Hyperparameter tuning	[56]
2021	Acute kidney injury	MLR	MLR	0.79	No	Not specified	UA, MLR	[58]
2022	Acute kidney injury	XGBoost	XGBoost	0.88	No	Literature review, clinical knowledge	Grid search optimisation	[37]
2022	Acute kidney injury	SVM, DT, RF, MLR, NN, AB, XGBoost, NB	NN	0.82	No	Clinical knowledge, previous studies	RF recursive feature elimination	[38]
2022	Acute kidney injury	MLR, elastic net, RF, SVM	SVM	0.74	No	Not specified	UA, MLR	[48]
2023	Acute kidney injury	MLR	MLR	0.77	No	Not specified	Stepwise selection	[45]
2023	Acute kidney injury	Catboost, GBM, RF	Catboost	0.82	Cumulative, slope, duration	Literature review, clinical knowledge	Recursive feature elimination	[43]
2024	Acute kidney injury	MLR, SVM, k-nearest neighbour, RF, XGBoost, lightgbm, Catboost	Catboost	0.82	Mean	Guidelines	Hyperparameter adjustment	[32]
2024	Acute kidney injury	RNN, gated recurrent unit	RNN	0.82	Max., min.	Not specified	Artificial NN	[49]
2022	Adverse drug event	MLR, GBM, RF, lasso, ridge, elastic net	GBM	0.75	No	Not specified	Hyperparameter tuning	[44]
2018	Bleeding risk	MLR	MLR	0.72	No	Not specified	BE	[42]
2022	Chemotherapy-induced ADR	MLR, DT, NN	MLR	0.67–0.83	No	Literature review	UA	[50]
2024	Coagulation disorder	MLR	MLR	0.73	Duration	Previous studies, clinical knowledge	UA, MLR	[46]
2023	Delirium	MLR	MLR	0.79	No	Literature review	Feature selection filter, forward and backward selection	[27]
2022	Liver injury	MLR	MLR	0.83	No	Not specified	Forward selection	[65]
2018	Fall risk	MLR	MLR	0.69	Unclear	Guidelines, literature review, clinical knowledge	UA, CA, fast BE, reduced BE, expert model	[22]
2024	Gastrointestinal side effects	MLR	MLR	0.84	No	Not specified	Stepwise selection	[66]
2024	Heart failure	XGBoost, RF	XGBoost	0.79	No	Not specified	UA, stepwise forward selection, Bayesian optimization for hyperparameter tuning	[47]
2017	Hyperkalaemia	GAM	GAM	0.84	All measurements	Not specified	Not specified	[55]
2024	Hyperthyroidism	LIR, RF, XGBoost, SVM	XGBoost	0.83	No	Not specified	LASSO	[28]
2017	Hypoglycaemia	MLR	MLR	0.73	No	Literature review	UA	[41]
2018	Hypoglycaemia	MLR	MLR	0.89	Trend, min., max.	Literature review	UA, CA, BE	[23]
2018	Hypoglycaemia	MLR	MLR	0.80	No	Clinical knowledge, previous studies	Trial and error, automated stepwise selection, evaluation of the information criteria	[33]
2020	Hypoglycaemia	MLR, RF, NN	XGBoost	0.96	Average	Clinical knowledge, previous studies	None	[51]
2021	Hypoglycaemia	MLR	MLR	0.71	No	Clinical knowledge	Statistical significance	[40]
2024	Hypoglycaemia	MLR, RF, XGBoost	MLR	0.81	Score	Previous studies	Not specified	[39]
2021	Hypoglycaemia	MLR, RF, NB, GBM	GBM	0.90	Mean, min., max., change	Clinical knowledge, Previous studies	UA, stepwise logistic regression, variable importance plots, relative influence plots	[26]
2024	Intracranial haemorrhage	MLR	MLR	0.95	No	Not specified	UA	[67]
2024	Major adverse cardiovascular event	RNN	RNN	0.98	Mean, time series analysis	All available	Extreme gradient boosting	[29]
2023	Major gastrointestinal bleeding	MLR	MLR	0.83	No	All available	UA, MLR	[68]
2020	Medication harm	MLR	MLR	0.72	No	Literature review, clinical knowledge	UA, chi-squared test, regression analyses	[25]
2017	Nephrotoxicity	DT	DT	Misclassification 13.2 + 1.4%	No	Literature review	UA, stepwise approach	[60]
2019	Nephrotoxicity	DT, MLR	DT	Misclassification 13.9 +- 1.7%	No	Literature review	UA, stepwise approach	[53]
2020	Nephrotoxicity	MLR	MLR	0.83	No	Literature review	UA	[61]
2020	Nephrotoxicity	NN, MLR	NN	0.83	No	Literature review	UA	[52]
2024	Nephrotoxicity	LASSO regression, SVM, NB, DT, RF, adaboost	RF	0.58	Cumulative number, AUC steady state	Literature review	Hyperparameter tuning	[54]
2022	Neurotoxicity	MLR	MLR	0.71	No	Literature review	LASSO, selection operator techniques	[69]
2019	Neutropenia	DT	DT	Misclassification 15.4% +- 1.8%	No	Literature review	MLR	[70]
2022	Neutropenia	MLR	MLR	0.93	No	Not specified	UA	[71]
2025	Osteonecrosis of the jaw	MLR, DT, RF, SVM, multilayer perceptron	MLR	0.89	No	Not specified	UA	[31]
2017	Preventable ADE	MLR	C-score	0.95	Min., change	Literature review, drug monographs, clinical knowledge	Clinical experts, CA, UA, fast BE, reduced back-step	[20]
2019	QT prolongation	MLR	MLR	0.83	No	Literature review, clinical knowledge, clinical knowledge	UA, CA, BE, reduced BE	[34]
2020	QT prolongation	ULR	risk score	0.59	No	Literature review	UA	[59]
2021	QT prolongation	NB, MLR, RF, NN	NN	0.71	No	All available	Not specified	[35]
2022	QT prolongation	DL, CA	DL, CA	0.78	No	Previous studies	Association analysis, max. information coefficient	[36]
2022	QTc prolongation	LIR	LIR	0.75	No	Literature review	Stepwise selection	[3]
2022	Respiratory depression	MLR	MLR	0.82	No	Literature review	LASSO	[72]
2025	Severe diarrhoea	MLR	MLR	0.79	No	Not specified	UA	[73]
2020	Severe opioid-related ADE	COX	COX	0.71	No	Clinical knowledge, previous studies	Statistical significance	[74]
2022	Thrombocytopenia	MLR	MLR	0.80	No	Not specified	UA	[62]
2022	Thrombocytopenia	MLR	MLR	0.80	No	All available	UA, BE	[63]
2023	Thrombocytopenia	MLR	MLR	0.84	No	All available	UA	[64]
2024	Venous thromboembolism	MLR	MLR	0.78	No	Clinical knowledge	Bivariate tests	[30]

Abbreviations: AB, Adaptive Boosting; ADR, Adverse Drug Reaction(s); AUC-ROC, Area Under the Curve of the Receiver Operating Characteristic; BE, Backward Elimination CA, Cluster Analysis; Catboost, Categorical Boosting; CBI, Combinatorial Inference; COX, Cox Proportional Hazard; DL, Deep Learning; DT, Decision Tree Model; GAM, Generalised Additive Model; GBM, Gradient Boosting Machine; LASSO, Least Absolute Shrinkage And Selection Operator; LIR, Linear Regression; MLR, Multivariable Logistic Regression; NB, Naive Bayes; NN, Neural Network; RF, Random Forest; SVM, Support Vector Machine; UA, Univariate Analysis; ULA, Univariate Logistic Regression; XGBoost, Extreme Gradient Boosting

Overall, the included studies developed prediction models for 28 different outcomes. The most frequently used outcomes were acute kidney injury [21, 24, 32, 37, 38, 43, 45, 48, 49, 56–58], hypoglycaemia [23, 26, 33, 39–41, 51], QT prolongation [3, 34–36, 59], nephrotoxicity [52–54, 60, 61], and thrombocytopenia [62–64]. Only one outcome (predicted QTc duration 24–48 h after onset of a drug-drug interaction) was predicted as a continuous variable whereas all other outcomes were binary or dichotomised from continuous variables. The number of observed ADR was highly variable covering data from 27 − 17,482 patients to 1,256-5,452 patient days or 466-2,727 events. It also varied between and within outcomes, e.g., 96.2 ± 28.3 patients with nephrotoxicity [52, 53, 60, 61] versus 2,943 ± 3,565 patients with hypoglycaemia [40, 41, 51].

**Fig. 1:**
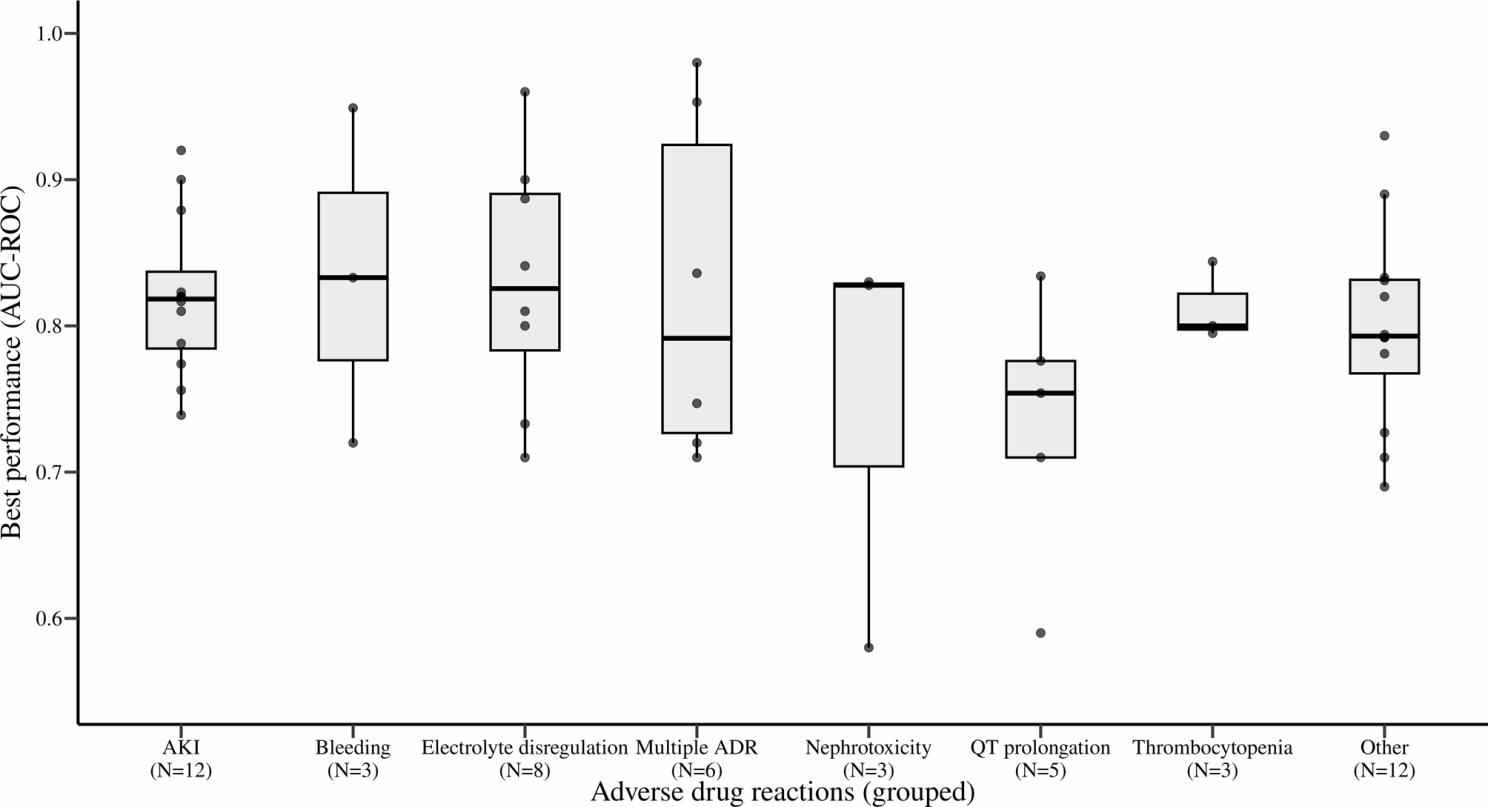
Model performance across different adverse drug reaction groups as extracted from the literature search (visualised as a box plot distribution). The group “Electrolyte dysregulation” consists of two outcomes, hypoglycaemia and hyperkalaemia, respectively. The group “Other” consist of all uniquely identified outcomes which could not be grouped. Abbreviations: AKI, Acute kidney injury; AUC-ROC, Area Under the Curve of the Receiver Operating Characteristic

For studies that reported more than one c-statistic (multiple models developed), the fit of the final model was considered. The largest area under the curve of the receiver operating characteristic (AUC-ROC) ranged from 0.58 to 0.98 (Fig. 1) and decision tree misclassification from 13.2 to 15.4%. Overall, in 59% of the studies the best model was a conventional model (logistic/linear regression, cox proportional hazard models, scores) but outperformed machine learning methods in only three (3/13) cases [31, 39, 50] where multiple models both with conventional and machine learning methods were developed. Two studies reported the development of hypothetical alert for monitoring reminders for potassium measurements [55] and warning of acute kidney injury risk [49].

Based on the findings of this review (identified outcomes, predictor selection methods, use of longitudinal information), we developed a conceptual framework for a Prescribing Monitoring system. It proposes how risks should be estimated individually, consider specific patient characteristics, and predict outcomes longitudinally over time. How predictions should be dynamically updated using population data and regularly reassess the accumulating data of the individual patient. The detailed steps of the framework, along with a use-case example (prediction of potassium levels) using simulated data, are provided in the Supplement.

## Discussion

In our scoping review that screened for predictive models to monitor drug-related adverse effects in hospitalised patients, we identified potential situations and clinically relevant outcomes where a longitudinal monitoring of patient risks (i.e., Prescribing Monitoring) appears clinically promising and technically feasible. In the following, we first summarise our main findings and compare them with related reviews that examined risk prediction models in EHR. We then explore how a Prescribing Monitoring system could enhance the potential of current prediction models and the challenges in the implementation.

Taking a closer look at how the predicted adverse effect outcomes have been defined and addressed in existing models is an important first step towards their potential use. When predicting ADRs, the focus should be on their preventability and on prioritizing actionable measures. Prediction models are most useful when they enable prescribers to act pre-emptively by warning them about ADR risks that can still be mitigated. However, Yasrebi-de Kom et al. [16] found in their review that only one study involved key stakeholders in identifying clinically relevant outcomes prior to model development. Nevertheless, in our study, most of the ADRs identified could be classified as *very important* or *important* based on the classifications proposed by Jeon et al. [75] and Haerdtlein et al. [76], even though the outcome selection process was not always explicitly described in the original publications. This suggests that, at least in the inpatient setting, the researchers often have an implicit understanding of which ADR outcomes are clinically relevant and potentially preventable.

After selecting a relevant outcome, researchers must consider which of the available EHR variables could effectively predict the risk of future ADRs. Unfortunately, many studies in this review failed to report how candidate predictors were chosen prior to model development. This lack of transparency makes it difficult to assess whether all potentially meaningful variables were considered from the EHR. Once potential predictors have been identified, the next critical step is to develop a parsimonious model that includes the most informative variables. This is typically achieved using statistical methods for predictor selection. This process is described in sufficient detail in most of the original papers, but some of the techniques are now considered outdated, such as the commonly used univariate analysis approach with pertinent limitations [17]. Specifically, it fails to account for how variables may act together in influencing the outcome. Predictors that seem unimportant when considered individually may become relevant when combined with other variables in a multivariate model, and vice versa [77].

A prerequisite for any predictor selection, however, is the availability and completeness of variables within the dataset. In practice, variables are often excluded prior to model development due to a high proportion of missing data. This can compromise model development, model validation, and model assessment [78]. Our findings are partly in line with previous reviews that found the insufficient reporting of how missing data were handled [16, 17]. We found that more than two-third of the studies included in our review addressed handling of missing data, employing techniques ranging from simple imputation to multiple imputation, indicating an increasing awareness of handling missing data.

The selection of predictors is also influenced by the form in which they are present in the data set. We found a consistent use of data sets to develop the prediction models as already described in similar reports [16, 17]. These datasets generally contain information collected over time, offering variables that reflect patient data across extended periods. However, prior reviews did not focus on the use of longitudinal information in the models. Here, our scoping review found that 78% of the studies did not include longitudinal predictors. As a result, important time-dependent factors such as cumulative drug exposure (e.g., in case of aminoglycoside-induced nephrotoxicity), the temporal effects of drug therapies (e.g., glucocorticoid-induced adrenal insufficiency), and the natural disease progression (e.g., worsening of stages of heart failure or increasing frailty index) could have been overlooked sometimes. Such temporal dynamics can substantially influence the likelihood of ADR [79, 80]. So, to summarise this aspect, our findings align with those of a review on EHR-based risk prediction models, which also reported a frequent neglect of using longitudinal information [81].

While we identified multiple potential use cases for a Prescribing Monitoring in our scoping review, the most commonly predicted adverse outcomes were acute kidney injury, electrolyte disorders, and QT prolongation. These events often develop rapidly during inpatient stays, leading to rapidly changing risk profiles that require frequent reassessment. For example, Kate et al. [11] emphasised the importance of continually updating AKI risk prediction, noting that static models may fail to reflect new risks that emerge during hospitalisation. We also found that only a few models were applied repeatedly during an inpatient stay, for example, models predicting the risk of hypoglycaemia during antihyperglycaemic therapy [23] or AKI during hospitalisation [24]. These findings underscore how challenging it is to implement a Prescribing Monitoring system within real-world EHR environments.

These fast-changing environments certainly affect when and how prediction models are used in clinical practice, particularly regarding high-risk predictions, resulting actions, and impact, i.e., the clinical utility of a prediction model. We found only few models with reported clinical utility. Of these, two studies included hypothetical alerts after their development: One compared their AKI alert to the implemented system [49], and another generated hypothetical potassium measurement reminder [55]. Techniques such as clinical impact curve analysis were only rarely applied, which suggests that integration into clinical workflows is still a long way off.

### Limitations

Based on the search strategy of a scoping review, the following facts may limit these results: As the main outcome was the prediction of ADR, other clinically relevant outcomes without being explicitly stated as ADR or drug-induced may not have been included. We did not include an optional quality assessment of the included studies, as direct comparisons were not performed and study quality was not considered to affect the conclusions drawn regarding the Prescribing Monitoring framework. We emphasise that the datasets differed between the studies, among many other things, all of which make it difficult to infer advantages between models being developed in such heterogeneous contexts. Despite these differences, we also highlighted commonly predicted ADRs and areas where more research is needed which can inform the development of Prescribing Monitoring systems. These systems are not meant to follow a single blueprint but should be tailored to specific outcomes, available data, and clinical workflows.

The implementation of such systems comes with several challenges, including differences in structures, data accessibility, and data integration (across institutions or wards). Some EHR contain unstructured data - such as progress notes, nursing documentation, and discharge summaries - that may be suitable for information extraction through natural language processing [82]. Building on such information, a Prescribing Monitoring system can enhance the potential of current prediction models in dynamically updating risk estimates based on accumulating patient data, and adapting prediction horizons and alerts to the specific clinical context of each ADR. In addition, where routine data may fall short, researchers can enrich their EHRs with missing predictors by prospective data collection to maximise the use of clinical data. Finally, this also implies, successful integration of these systems into CDSS require supplementary input from healthcare professionals to address (data) gaps.

To this end, this scoping review and drafted framework point out critical steps needed to understand the variables in the EHR, identify potential predictors and their operationalisation to support the monitoring of ADR in the inpatient setting.

## Conclusion

In the last decade, research has successfully focused on models predicting preventable and clinically relevant ADR. However, as risks may fluctuate, the efforts should be intensified to consider also longitudinal predictors and establish dynamic outcome predictions that are relevant to successfully implement Prescribing Monitoring into a CDSS. On the basis this scoping review, researchers will be able to make better-informed plans in future, in particular with regard to proper outcome selection, predictor operationalisation, and longitudinal modelling to dynamically predict individual risks. This could improve the use of real-world data in clinical routine and help to improve the appropriateness of drug therapies and medication safety.

## Electronic supplementary material

Below is the link to the electronic supplementary material.

Supplementary Material 1: Additional file 1: Includes Tables and figures for the description of the scoping review methodology, supporting tables, figures, and code for the Prescribing Monitoring framework

Supplementary Material 2: Additional file 2: Includes the simulated fixed and temporal dataset to reproduce the results of the Prescribing Monitoring example

## Data Availability

The simulated data is available in the Supplementary Materials; further inquiries can be directed to the corresponding authors.

## Abbreviations

AB: Adaptive Boosting
ADR: Adverse Drug Reaction
BE: Backward Elimination
CA: Cluster Analysis
CatBoost: Categorical Boosting
CBI: Combinatorial Inference
CDSS: Clinical Decision Support System
COX: Cox Proportional Hazard
DL: Deep Learning
DT: Decision Tree Model
EHR: Electronic Health Record(s)
GAM: Generalised Additive Model
GBM: Gradient Boosting Machine
LASSO: Least Absolute Shrinkage And Selection Operator
LIR: Linear Regression
MLR: Multivariable Logistic Regression
NB: Naive Bayes
NN: Neural Network
PRISMA-ScR: Preferred Reporting Items for Systematic Reviews and Meta-Analyses Extension for Scoping Reviews
RF: Random Forest
RWE: Real-world Evidence
SGB: Stochastic Gradient Boosting
SVM: Support Vector Machine
UA: Univariate Analysis
ULA: Univariate Logistic Regression
XGBoost: Extreme Gradient Boosting
