# Supplementary material for: Towards a prescribing monitoring system for medication safety evaluation within electronic health records: a scoping review
Source: BMC Med Inform Decis Mak. 2025 Jul 2;25:244. doi: 10.1186/s12911-025-03096-3 (PMC12224581; doi:10.1186/s12911-025-03096-3)
Supplement: Supplementary file 1 — Supplementary Material 1: Additional file 1: Includes Tables and figures for the description of the scoping review methodology, supporting tables, figures, and code for the Prescribing Monitoring framework [file 12911_2025_3096_MOESM1_ESM.docx]

**Supplementary Material**

**Development of a Prescribing Monitoring system framework in an EHR**

The scoping review identified clinically relevant adverse drug reactions in the inpatient setting, most of which could be predicted with satisfactory accuracy. All ADR were clinically potentially serious complications that can lead to deterioration and prolongation of an inpatient stay. However, less than a quarter of the proposed models included longitudinal variables in a patient setting where changes in predictors occur regularly. Consequently, we propose a strategy for the development of an inpatient Prescribing Monitoring system to predict specific ADR. This established framework will be used as a strategic plan for conducting further studies based on the following steps (Table S4).

**Table S4: Framework steps for the development of a Prescribing Monitoring system**

| Proposed steps | Considerations |
| --- | --- |
| 1. Identification of clinically relevant outcomes | - Identify what potential outcomes can be extracted from the EHR. - Focus on preventable outcomes [1, 2] and clinically important ADR [3, 4]. - The outcome should be clearly definable and algorithmically identifiable in the EHR data. |
| 1. Definition of the patient cohort | - Define the population of interest, patient setting, potential predictors, and index times. - Identify the risk horizon relevant to the patient (e. g., risk on admittance, during the inpatient stay, after discharge). |
| 1. Identification of candidate predictors prior to model development | - Extraction of candidate predictors through literature review and clinical knowledge. - Are they extractable from the EHR (e. g., potential difficulties in extracting information such as disease stages or frailty index) and can proxy variables (e. g., surrogate markers) be used? |
| 1. Initial data analysis | - Characterisation of distribution, completeness, collinearities, and associations between the potential predictors. - STRATOS group framework on initial data analysis for longitudinal studies [5]. |
| 1. Operationalisation of variables for longitudinal models | - Processing of longitudinal data using all measurements, best singe measurement, summary measurements, trajectories, changes between measurements, conditional measurements (regression of all previous measurements on a specific time point of a measurement), or growth curve parameters [6]. - Transformation into usable format, the so-called time-series data preparation (Figure S3) [7]. |
| 1. Model development | - Use of suitable statistical models for longitudinal data analysis (e. g., mixed-effects model such as generalised additive mixed-effects models). - Handling of irregularly spaced measurements (e. g., by applying different imputation methods or suitable statistical models). |
| 1. Validation | - Out-of-sample validation (e. g., in split, external, or bootstrapped data). - Performance metrics e. g., area under the curve, misclassification rates, or root mean-squared error [8]. |

In the remainder of this part, we focus on the theoretical development of a prediction model for potassium values in simulated data in order to identify patients at risk for hyperkalaemia (the same model could be used to predict the risk of hypokalaemia). In this use case, we define the outcome as the maximum potassium value in the next 24 h during an inpatient stay, which can be predicted longitudinally and dynamically with updated information on conditions, prescriptions, and laboratory values. The model would focus on a typical inpatient population and exclude more complex patients such as paediatric patients, dialysis patients, intensive care patients, or emergency visits. Additionally, patients with missing baseline characteristics, or less than two potassium measurements would be excluded from model development (Figure S2). Potential candidate predictors (Table S5) were identified by literature search (studies on risk factors [9, 10]) and clinical knowledge (DC, expert in clinical pharmacology, internal medicine, and nephrology; WEH, expert in clinical pharmacology and internal medicine).

To illustrate the steps 5-7 in Table S4, we provide an example data set (Supplementary Material) with static and dynamic variables to be used for variable operationalisation and statistical modelling. The example dataset consists of 500 virtual patients with multiple potassium measurements recorded during their hospital stay. The measurements are irregularly spaced and include gaps during the stay to mimic the data structure found in real EHR. Upon admission, they randomly start on 0-3 prescriptions that can affect their potassium values (Code S1). The model aims to predict for each patient the maximum serum potassium concentration for the next 24 h. The prediction model uses three static predictors (start of the new prescriptions 1-3) and dynamic predictors (maximum potassium value 24, 48, and 72 h before the index time). For the first 48 h after admission, we developed three separate models (gam_mod0/24/48, Code S1). After 72 h, all information for the final model was available and included (gam_mod72). Missing longitudinal predictors were substituted by the previous predicted potassium maximum. This enabled a continuous prediction over the complete duration of each patient’s hospital stay (see Supplemental Figure S4 for details). For the simpler presentation in our use case, we have evaluated the performance in-sample (Figure S4 (c) and (d)) with a root mean square error of 0.455, an average fold error (AFE) of 1.02, an absolute average fold error (AAFE) of 1.14, and a percent prediction error (PPE) of 5.62. Model predictions were deemed satisfactory (for 0.8 ≤ AFE ≤ 1.25 and AAFE ≤ 1.25, where lower values of PPE indicate better prediction [11]). The satisfactory performance of the model could be expected from analysing the simulated data. Nevertheless, it provides a proof-of-principle that the suggested methodological approach is suitable to derive the necessary information also from any identically structured data set.

**Table S1.** PRISMA-ScR Checklist.

| **Section** | **Line** |
| --- | --- |
| **Title** | ll. 1-2 |
| **Abstract** | |
| Structured summary | ll. 51-79 |
| **Introduction** |  |
| Rationale | ll. 104-112 |
| Objectives | ll. 113-116 |
| **Methods** | |
| Protocol and registration | ll. 121-122 |
| Eligibility criteria | ll. 133-138 and Table S3 |
| Information sources | l. 145 |
| Search | ll. 139-147 and Table S2 |
| Selection of sources of evidence | ll. 148-154 |
| Data charting process | ll. 154-156 |
| Data items | ll. 157-158 |
| Critical appraisal of individual sources of evidence | optional, not necessary for the scope of this article. |
| Summary measures | Not applicable for scoping reviews. |
| Synthesis of results | ll. 159-164 |
| Risk of bias across studies | Not applicable for scoping reviews. |
| Additional analyses | Not applicable for scoping reviews. |
| **Results** | |
| Selection of sources of evidence | l. 167 and Figure S1 |
| Characteristics of sources of evidence | ll. 167-168 and Table 1 |
| Critical appraisal within sources of evidence | optional, not necessary for the scope of this article |
| Results of individual sources of evidence | Table 1 |
| Synthesis of results | ll. 166-209 and Table 1 |
| Risk of bias across studies | Not applicable for scoping reviews. |
| Additional analyses | Not applicable for scoping reviews. |
| **Discussion** | |
| Summary of evidence | ll. 213-275 |
| Limitations | ll. 276-286 |
| Conclusion | ll. 301-308 |
| **Funding** | l. 318 |

**Table S2.** Database search strategies.

| **Database** | **Search term** |
| --- | --- |
| **MEDLINE** | ((humans[Filter] AND (patient OR patients))  AND  (((predict[tiab]) OR (prediction[tiab]) OR (predicting[tiab]) OR (predicted[tiab]) OR predictive[tiab])) AND ((risk prediction model[tw]) OR (prediction model[tw]) OR (monitoring[tw]) OR (model[tw]) OR (models[tw]) OR (modelling[tw]) OR (modelled[tw]))) AND ((pred*[ti]) OR (mode*[ti]) OR (risk[ti]) AND ((drug*[tiab]) OR (medication*[tiab]) OR (polypharmacy*[tiab])))  AND  ((adverse drug reaction) OR (adverse effect) OR (side effect) OR (drug interaction) OR (drug adverse effect) OR (adverse drug event) OR (drug side effect) OR (drug-related adverse event) OR (drug-induced[tiab]) OR (chemically induced[tiab]) OR (drug related[tiab]) OR (Drug interactions[MeSH Terms]) OR (drug-associated[tiab]))  NOT  ((Clinical Trial, Phase I[Publication Type]) OR (Clinical Trial, Phase II[Publication Type]) OR (Clinical Trial, Phase III[Publication Type]) OR (multicenter study[Publication Type]) OR (genomic) OR (review[Publication Type]) OR (Meta-Analysis[Publication Type]))  AND  ((english[Filter] OR german[Filter]) AND (2013:2025[pdat])) |
| **EMBASE** | 'patient'  AND  (predict:ab,ti OR prediction:ab,ti OR predicting:ab,ti OR predicted:ab,ti OR predictive:ab,ti) AND ('risk prediction model':ti,ab,kw OR 'monitoring':ti,ab,kw OR 'model':ti,ab,kw OR 'models':ti,ab,kw) AND (pred*:ti OR mode*:ti OR risk:ti)  AND  (‘adverse effect' OR 'side effect' OR 'drug interaction' OR 'adverse drug reaction' OR 'drug induced':ab,ti OR 'drug interaction'/exp OR 'drug associated':ab,ti) AND (drug*:ab,ti OR medication*:ab,ti OR polypharmacy*:ab,ti)  NOT  ('phase 1 clinical trial'/exp OR 'phase 2 clinical trial'/exp OR 'phase 3 clinical trial'/exp OR 'multicenter study'/exp OR 'genomics' OR 'review'/exp OR 'meta analysis'/exp)  AND  [2013-2025]/py AND ([english]/lim OR [german]/lim) |

**Table S3.** Eligibility criteria for the scoping review of prediction models for adverse drug reactions published between 2013 to May 2025.

| **Stages** | **Criteria** | **Inclusion** | **Exclusion** |
| --- | --- | --- | --- |
| All stages | Population | - Human - Inpatients | - Animal - Outpatients |
| All stages | Setting | - Hospital (any ward) - Use of electronic health records, clinical physician order entry or CDSS | - Settings other than hospital - Non-computerised patient recording |
| All stages | Intervention | - Prediction models for adverse drug reactions | - Prediction models for diagnosis or other not related to adverse drug reactions - Prediction of allergy reactions |
| All stages | Language | - English - German | - Other languages |
| Full-text screening | Intervention | - Reporting statistical model | - No report on statistical model |
| Full-text screening | Outcome | - Reporting of model performance | - No reports on model performance |
| Full-text screening | Other | - Full text available | - Only abstract available |

**Table S5.** Preliminary list of candidate predictors for the prediction of potassium values.

| **Predictors** | **Coding** |
| --- | --- |
| Potential potassium-elevating factors | |
| Co-morbidities | ICD-10 code |
| Chronic tubulo-interstitial nephritis | N11* |
| Non-dialysis renal disease | N17*, N18* (except N18.5), N19* |
| Haemolysis | D59.18, D59.8, D59.9 |
| Anaemia | D57*, D58*, D59 |
| Gastrointestinal bleeding | K92.2 |
| Rhabdomyolysis | T79.69 |
| Tumorlysis syndrome | E88.3 |
| Sickle-cell anaemia | D57* |
| Addison’s disease | E27.1 |
| Renal impairment | Estimated eGFR |
| Drugs | ATC-code |
| Potassium-sparing diuretics | C03D |
| Angiotensin-converting enzyme inhibitors | C09A, C09B |
| Angiotensin receptor blockers | C09C, C09D |
| Renin inhibitors | C09XA |
| NSAID incl. COX-2 inhibitor | M01A |
| Heparin | B01AB (except B01AB02, B01AB09) |
| Cotrimoxazole | J01EE |
| Calcineurin inhibitors | L04AD |
| Suxamethonium | M03AB01 |
| Potassium supplements | A12BA, B05XA01, B05XA30 |
| Potential potassium-decreasing factors | |
| Co-morbidities | ICD-10 codes |
| Polyuria | R35.0 |
| Acute diarrhoea | A00.*, A02.0, A03.*, A08.*, A09* |
| Chronic diarrhoea | K52.9 |
| Chronic vomiting | O21.1, F50.2, F50.5 |
| Cushing’s syndrome | E24* |
| Hyperaldosteronism, Conn’s syndrome | E26* |
| Hypomagnesaemia | Measured value < 0.75 mmol/l |
| Hypothermia | T68*, R68.0 |
| Drugs | ATC-codes |
| Low-ceiling diuretics | C03A, C03B |
| High-ceiling diuretics | C03C |
| Acetazolamide | S01EC01 |
| Osmotic diuretics (mannitol) | B05BC01 |
| Laxatives | A06A |
| Insulin | A10A |
| Glucocorticoids | H02AB |
| Amphotericin B | J02AA01 |
| Cisplatin | L01XA01 |
| Drugs for treatment of hyperkalaemia | V03AE01, V03AE09, V03AE10, V03AE11, |
| Exclusion | |
| Dialysis | T82.4, Y60.2, Y61.2, Y62.2, Y84.1, Z49.1, Z49.2, Z99.2, N18.5 |

NSAID, Non-steroidal anti-inflammatory drug; COX-2, Cyclooxygenase-2

**Table S5:** Timeline of the scoping review process.

| **Review Stages** | **Timeline** |
| --- | --- |
| Definition of objective | November 2022 |
| Definition of inclusion and exclusion criterias | November – Dezember 2022 |
| Preliminary search in MEDLINE and Google Scholar | January 2023 |
| Development of search strategy | Februrary – April 2023 |
| First run of search string in MEDLINE and EMBASE | May 2023 |
| Registration of scoping review protocol | June 16^th^ 2023 |
| First title and abstract screening | May – June 2023 |
| Development of extraction form | May – June 2023 |
| First full text screening | June – July 2023 |
| First data extraction | July 2023 – December 2024 |
| Second run of search string in MEDLINE and EMBASE | May 2025 |
| Second title and abstract screening | May 2025 |
| Second full text screening | May 2025 |
| Second data extraction | May 2025 |
| Data synthesis | May – June 2025 |

**
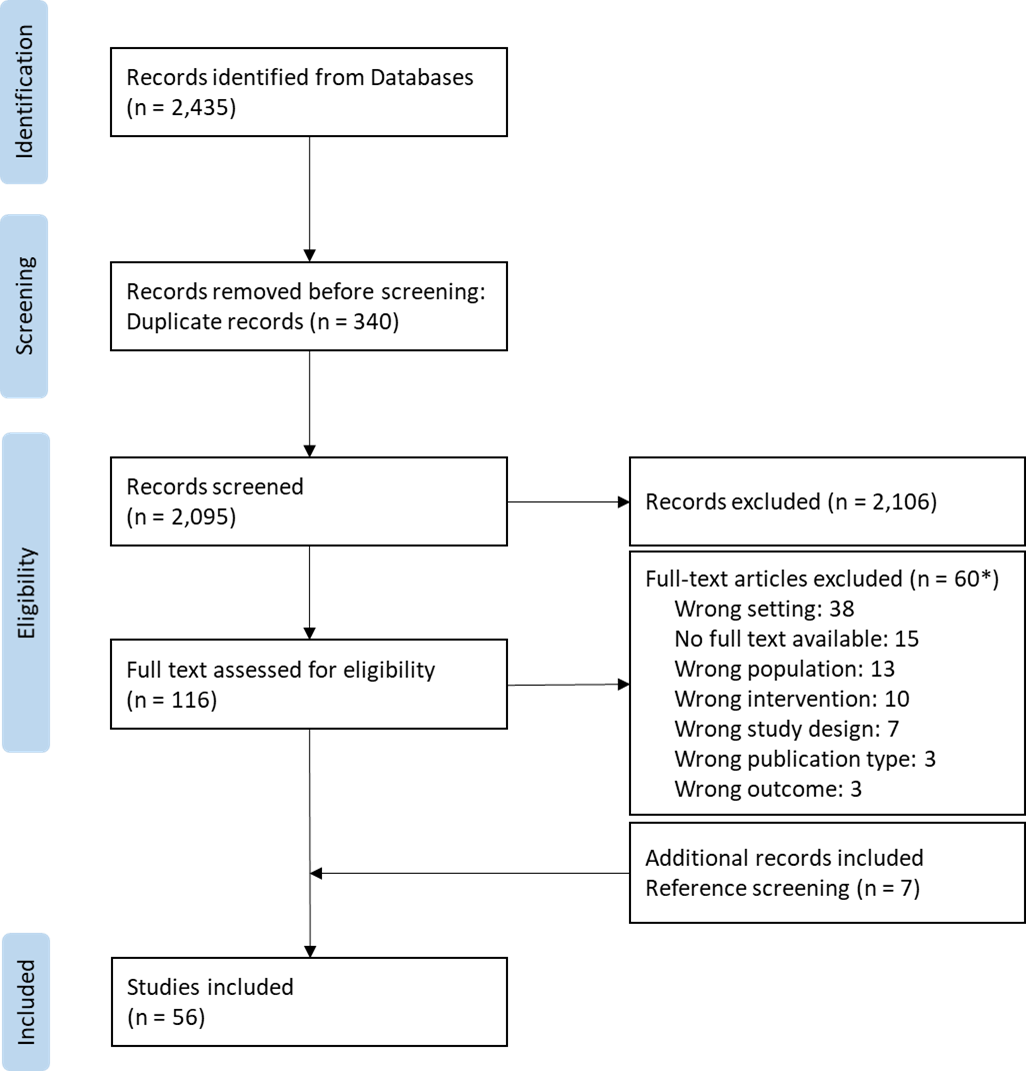
**

**Figure S1.** PRISMA flow diagram of records search and selection strategy.

***** Multiple entries were permitted, which explains why the sum of the reasons for exclusion exceeds the total number of articles


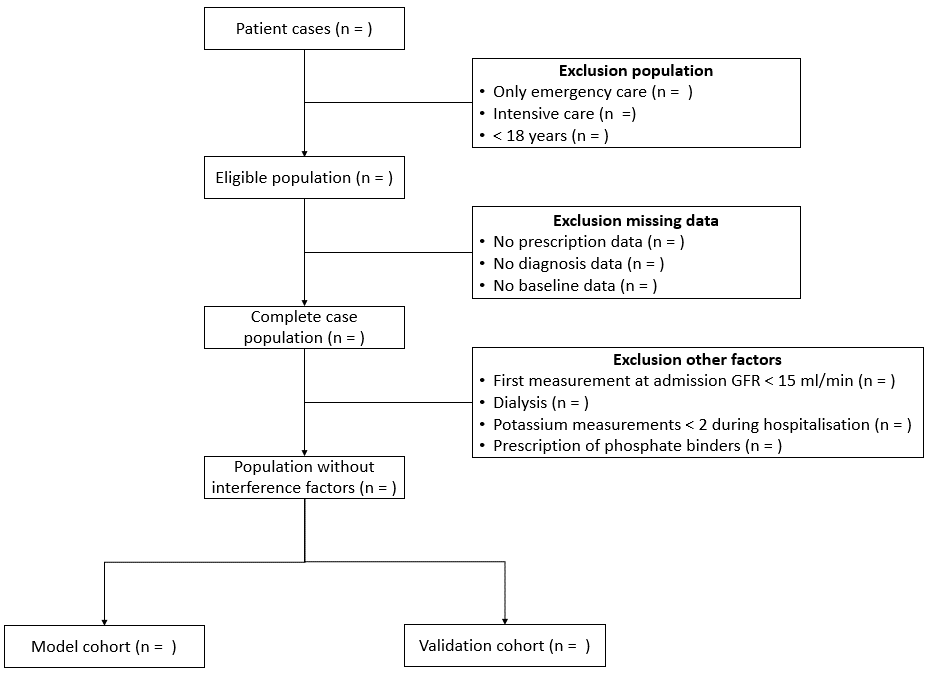


**Figure S2.** Exemplary patient flow chart to select the target population of a prediction model of potassium values (in terms of a development cohort and a split cohort for model validation).


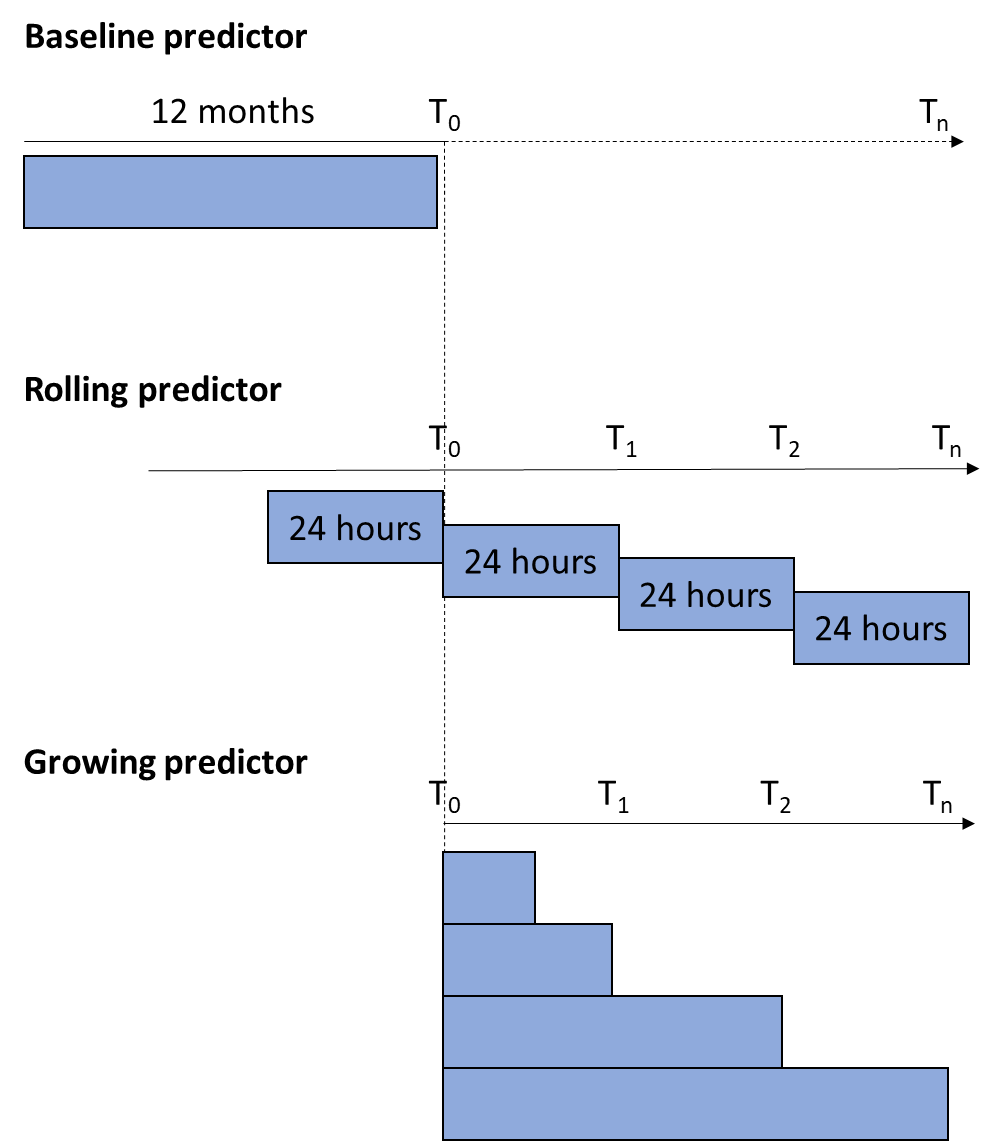


**Figure S3.** Operationalisation of variables in time-series data preparation (adapted from Meyer (2021) [7] (p. 66)). The data preparation of this time series enables the use of the experimental package gpmodels – A Grammar of Prediction Models (https://github.com/ML4LHS/gpmodels.git). Herein, the time stamps are transformed into indices, variable values are grouped and summarised by their index times, and long data formats are transformed to wide format with one row per index or period.

T, Time


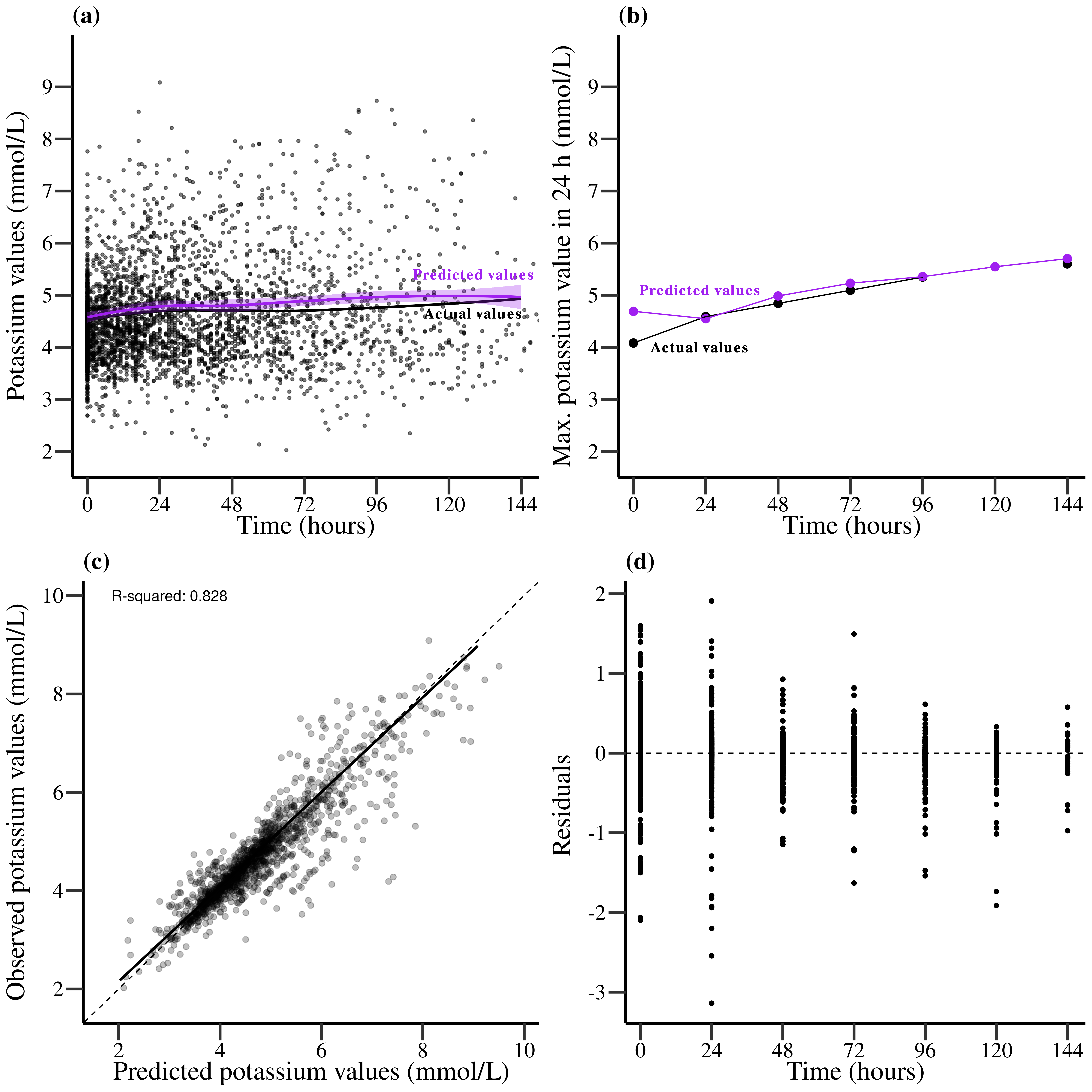


**Figure S4.** Model diagnostics for the prediction of the next maximum 24-h potassium values in the simulated data. **(a)** Simulated potassium measurements over the course of the inpatient stay. The trend lines are loess fits of the actual maximum values and the predicted potassium values in the next 24 h (black and purple line, respectively, with a 95 % confidence interval as a purple area). **(b)** Single simulated patient course of observed maximum potassium values in the next 24 h (black) and predicted values (purple). **(c)** Observed (i. e., actually simulated) vs. predicted values with a linear regression (black line) and dotted 1:1 line. **(d)** Residuals (mmol/L) over time for all predicted to observed values.

**Code S1.** Reproducible code snippet for the potassium value prediction model.

*if(!require("tidyverse")) {install.packages("tidyverse"); library("tidyverse", quietly = TRUE)}*

*if(!require("here")) {install.packages("here"); library("here", quietly = TRUE)}*

*if(!require("gamm4")) {install.packages("gamm4"); library("gamm4", quietly = TRUE)}*

*if(!require("ggplot2")) {install.packages("ggplot2"); library("ggplot2", quietly = TRUE)}*

*if(!require("geomtextpath")) {install.packages("geomtextpath"); library("geomtextpath", quietly = TRUE)}*

*if(!require("gpmodels")) {remotes::install_github('ML4LHS/gpmodels'); library("gpmodels", quietly = TRUE)}*

*if(!require("msir")) {install.packages("msir"); library("msir", quietly = TRUE)}*

*if(!require("gridExtra")) {install.packages("gridExtra"); library("gridExtra", quietly = TRUE)}*

*# load df fixed and temporal*

*sample_fixed_data_sim <- read.csv2(here("sample_fixed_data_sim.csv"))*

*sample_temporal_data_sim <- read.csv2(here("sample_temporal_data_sim.csv"))*

*sample_fixed_data_sim <- sample_fixed_data_sim %>%*

*mutate(admit_time = stringr::str_replace_all(admit_time, "T", " ")) %>%*

*mutate(admit_time = as.POSIXct(stringr::str_replace_all(admit_time, "Z", " "))) %>%*

*mutate(dc_time = stringr::str_replace_all(dc_time, "T", " ")) %>%*

*mutate(dc_time = as.POSIXct(stringr::str_replace_all(dc_time, "Z", " ")))*

*sample_temporal_data_sim <- sample_temporal_data_sim %>%*

*mutate(time = stringr::str_replace_all(time, "T", " ")) %>%*

*mutate(time = stringr::str_replace_all(time, "Z", " ")) %>%*

*mutate(time = as.POSIXct(time))*

*# gpmodels training set ----------------------------------------------------------------*

*n_patients <- 500*

*n_days <- 7*

*future::plan('multisession')*

*unlink(file.path(tempdir(), 'gpmodels_dir', '*.*'))*

*tf = time_frame(fixed_data = sample_fixed_data_sim,*

*temporal_data = sample_temporal_data_sim %>% dplyr::filter(id %in% 1:n_patients),*

*fixed_id = 'id',*

*fixed_start = 'admit_time',*

*fixed_end = 'dc_time',*

*temporal_id = 'id',*

*temporal_time = 'time',*

*temporal_variable = 'variable',*

*temporal_category = 'category',*

*temporal_value = 'value',*

*step = hours(24),*

*max_length = days(7),*

*output_folder = file.path(tempdir(), 'gpmodels_dir'),*

*create_folder = TRUE)*

*# gpmodels predictors and outcomes ----------------------------------------*

*tf %>%*

*add_rolling_predictors(variables = 'K',*

*lookback = hours(72),*

*window = hours(24),*

*stats = c(max = max),*

*impute = FALSE) %>%*

*add_baseline_predictors(variables = 'K',*

*lookback = hours(48),*

*window = hours(48),*

*offset = hours(-48),*

*stats = c(min = min)) %>%*

*add_rolling_outcomes(variables = 'K',*

*lookahead = hours(24),*

*stats = c(max = max))*

*model_data = combine_output(tf)*

*# save(model_data_GAM, file = here("data", "model_data_GAM.rda"))*

*# GAM --------------------------------------------------------------------*

*gam_mod0 <- gamm4(outcome_K_max_24 ~ time + StartPres1 + StartPres2 + StartPres3 + baseline_K_min_48,*

*family = gaussian,*

*data = model_data,*

*random = ~ (1|id),*

*REML = FALSE)*

*gam_mod24 <- gamm4(outcome_K_max_24 ~ time + StartPres1 + StartPres2 + StartPres3 + baseline_K_min_48 + K_max_24 ,*

*family = gaussian,*

*data = model_data,*

*random = ~ (1|id),*

*REML = FALSE)*

*gam_mod48 <- gamm4(outcome_K_max_24 ~ time + StartPres1 + StartPres2 + StartPres3 + baseline_K_min_48 + K_max_24 + K_max_48,*

*family = gaussian,*

*data = model_data,*

*random = ~ (1|id),*

*REML = FALSE)*

*gam_mod72 <- gamm4(outcome_K_max_24 ~ time + StartPres1 + StartPres2 + StartPres3 + baseline_K_min_48 + K_max_24 + K_max_48 + K_max_72,*

*family = gaussian,*

*data = model_data,*

*random = ~ (1|id),*

*REML = FALSE)*

*GAM_predict <- model_data %>%*

*mutate(fit = ifelse(time == 0, predict(gam_mod0$gam, model_data,se.fit = TRUE,type='response',*

*na.action=na.pass)$fit, NA)) %>%*

*mutate(fit = ifelse(time == 24, predict(gam_mod24$gam, model_data,se.fit = TRUE,type='response',*

*na.action=na.pass)$fit, fit)) %>%*

*mutate(fit = ifelse(time == 48, predict(gam_mod48$gam, model_data,se.fit = TRUE,type='response',*

*na.action=na.pass)$fit, fit)) %>%*

*mutate(fit = ifelse(time >= 72, predict(gam_mod72$gam, model_data,se.fit = TRUE,type='response',*

*na.action=na.pass)$fit, fit)) %>%*

*mutate(se.fit = ifelse(time == 0, predict(gam_mod0$gam, model_data,se.fit = TRUE,type='response',*

*na.action=na.pass)$se.fit, NA)) %>%*

*mutate(se.fit = ifelse(time == 24, predict(gam_mod24$gam, model_data,se.fit = TRUE,type='response',*

*na.action=na.pass)$se.fit, se.fit)) %>%*

*mutate(se.fit = ifelse(time == 48, predict(gam_mod48$gam, model_data,se.fit = TRUE,type='response',*

*na.action=na.pass)$se.fit, se.fit)) %>%*

*mutate(se.fit = ifelse(time >= 72, predict(gam_mod72$gam, model_data,se.fit = TRUE,type='response',*

*na.action=na.pass)$se.fit, se.fit))*

*# manage missing prediction ----------------------*

*GAM_interation <- GAM_predict*

*for(i in 1:n_days) {*

*GAM_interation <- GAM_interation %>%*

*mutate(K_max_24 = ifelse(is.na(fit) & is.na(K_max_24), lag(fit), K_max_24)) %>%*

*mutate(K_max_48 = ifelse(is.na(fit) & is.na(K_max_48), lag(K_max_24), K_max_48)) %>%*

*mutate(K_max_72 = ifelse(is.na(fit) & is.na(K_max_72), lag(K_max_48), K_max_72))*

*GAM_interation <- GAM_interation %>%*

*mutate(fit = ifelse(time == 24, predict(gam_mod24$gam, GAM_interation,se.fit = TRUE,type='response',*

*na.action=na.pass)$fit, fit)) %>%*

*mutate(fit = ifelse(time == 48, predict(gam_mod48$gam, GAM_interation,se.fit = TRUE,type='response',*

*na.action=na.pass)$fit, fit)) %>%*

*mutate(fit = ifelse(time >= 72, predict(gam_mod72$gam, GAM_interation,se.fit = TRUE,type='response',*

*na.action=na.pass)$fit, fit)) %>%*

*mutate(se.fit = ifelse(time == 0, predict(gam_mod0$gam, GAM_interation,se.fit = TRUE,type='response',*

*na.action=na.pass)$se.fit, NA)) %>%*

*mutate(se.fit = ifelse(time == 24, predict(gam_mod24$gam, GAM_interation,se.fit = TRUE,type='response',*

*na.action=na.pass)$se.fit, se.fit)) %>%*

*mutate(se.fit = ifelse(time == 48, predict(gam_mod48$gam, GAM_interation,se.fit = TRUE,type='response',*

*na.action=na.pass)$se.fit, se.fit)) %>%*

*mutate(se.fit = ifelse(time >= 72, predict(gam_mod72$gam, GAM_interation,se.fit = TRUE,type='response',*

*na.action=na.pass)$se.fit, se.fit))*

*}*

*GAM_final_data <- GAM_interation %>% dplyr::select(id, outcome_K_max_24, fit, se.fit, time)*

*# Definition of ggplot variables ------------------------------------------*

*axistext = 18*

*axistitletext = 22*

*axisline = 1.1*

*axisticks = 1.1*

*axistickslenght = 0.3*

*titlesize = 20*

*# Gam loess plot ---------------------------------------------------------*

*potassium_values <- sample_temporal_data_sim %>%*

*dplyr::filter(id %in% 1:n_patients) %>%*

*dplyr::select(id, time, value) %>%*

*group_by(id) %>%*

*mutate(time = as.integer(difftime(time, min(time), unit = "hours")))*

*Figure3A <- GAM_final_data %>%*

*ggplot() +*

*ggtitle("3A") +*

*geom_point(data = potassium_values,aes(y = value, x = time), size = 1, color = "black", alpha = 0.5) +*

*stat_smooth(method = "loess" , aes(y = outcome_K_max_24, x = as.numeric(time)),*

*colour = "black", fill = "black", alpha = 0.2, se = FALSE) +*

*stat_smooth(method = "loess" , aes(y = fit, x = as.numeric(time)), colour = "purple", fill = "purple", alpha = 0.3) +*

*theme_classic() +*

*scale_x_continuous("Time (hours)", limits = c(-5,150), breaks = seq(0,144, 24), expand=c(0,0)) +*

*scale_y_continuous("Potassium values (mmol/L)", limits = c(1.5,10), breaks = seq(0, 9.5, 1), expand=c(0,0)) +*

*theme(text = element_text(family = "serif"),*

*plot.title = element_text(face = "bold", size = titlesize),*

*axis.text.y = element_text(size = axistext, color = "black"),*

*axis.text.x = element_text(size = axistext, color = "black"),*

*axis.title.x = element_text(size = axistitletext, margin = margin(r = 13)),*

*axis.title.y = element_text(size = axistitletext, margin = margin(r = 10)),*

*axis.line = element_line(size = axisline),*

*axis.ticks = element_line(size = axisticks),*

*axis.ticks.length = unit(0.5, "cm"),*

*legend.title = element_blank(),*

*legend.text = element_text(size = 20),*

*legend.position = "top") +*

*geomtextpath::geom_textsegment(data = GAM_final_data, aes(x = 122, y = 5.4, xend = 142, yend = 5.4, label = "Predicted values"),*

*color="purple", size=4.4, inherit.aes = FALSE, family = "serif", spacing =-60, text_only = TRUE, fontface = "bold") +*

*geomtextpath::geom_textsegment(data = GAM_final_data, aes(x = 122, y = 4.65, xend = 142, yend = 4.65, label = "Actual values"),*

*color="black", size=4.4, inherit.aes = FALSE, family = "serif", spacing =-60, text_only = TRUE, fontface = "bold")*

*# individuals values max 24h potassium against fitted values --------------------------------*

*Figure3B <- GAM_final_data %>% filter(id == "370") %>%*

*ggplot() +*

*ggtitle("3B") +*

*geom_point(aes(y = outcome_K_max_24, x = time), size = 3, color = "black", alpha = 1) +*

*geom_point(aes(y = fit, x = time), size = 3, color = "purple", alpha = 1) +*

*geom_line(aes(y = outcome_K_max_24, x = time), color = "black", alpha = 1) +*

*geom_line(aes(y = fit, x = time), color = "purple", alpha = 1) +*

*theme_classic() +*

*scale_x_continuous("Time (hours)", limits = c(-5,150), breaks = seq(0,144, 24), expand=c(0,0)) +*

*scale_y_continuous("Max. potassium value in 24 h (mmol/L)", limits = c(1.5,10), breaks = seq(0, 9.5, 1), expand=c(0,0)) +*

*theme(text = element_text(family = "serif"),*

*plot.title = element_text(face = "bold", size = titlesize),*

*axis.text.y = element_text(size = axistext, color = "black"),*

*axis.text.x = element_text(size = axistext, color = "black"),*

*axis.title.x = element_text(size = axistitletext, margin = margin(r = 13)),*

*axis.title.y = element_text(size = axistitletext, margin = margin(r = 10)),*

*axis.line = element_line(size = axisline),*

*axis.ticks = element_line(size = axisticks),*

*axis.ticks.length = unit(0.5, "cm")) +*

*geomtextpath::geom_textsegment(data = GAM_final_data, aes(x = 12, y = 5, xend = 32, yend = 5, label = "Predicted values"),*

*color="purple", size=4.4, inherit.aes = FALSE, family = "serif", spacing =-60, text_only = TRUE, fontface = "bold") +*

*geomtextpath::geom_textsegment(data = GAM_final_data, aes(x = 12, y = 4, xend = 32, yend = 4, label = "Actual values"),*

*color="black", size=4.4, inherit.aes = FALSE, family = "serif", spacing =-60, text_only = TRUE, fontface = "bold")*

*# observed vs predicted -----------------------------------------------------------*

*r_squared <- summary(lm(outcome_K_max_24 ~ fit, data = GAM_final_data))$r.squared*

*Figure3C <- GAM_final_data %>%*

*ggplot() +*

*ggtitle("3C") +*

*geom_point(aes(x = fit, y = outcome_K_max_24), size = 2, alpha = 0.25, color = "black") +*

*scale_x_continuous("Predicted potassium values (mmol/L)", limits = c(1.3,10.3), breaks = seq(0,10, 2), expand=c(0,0)) +*

*scale_y_continuous("Observed potassium values (mmol/L)", limits = c(1.3,10.3), breaks = seq(0, 10, 2), expand=c(0,0)) +*

*geom_smooth(aes(x = outcome_K_max_24, y = fit), method = "lm", se = FALSE, color = "black") +*

*geom_abline(slope = 1, linetype = "dashed") +*

*annotate("text", x = 3, y = 10, label = paste("R-squared:", round(r_squared, 3)), size = 4.4) +*

*theme_classic() +*

*theme(text = element_text(family = "serif"),*

*plot.title = element_text(face = "bold", size = titlesize),*

*axis.text.y = element_text(size = axistext, color = "black"),*

*axis.text.x = element_text(size = axistext, color = "black"),*

*axis.title.x = element_text(size = axistitletext, margin = margin(r = 13)),*

*axis.title.y = element_text(size = axistitletext, margin = margin(r = 10)),*

*axis.line = element_line(size = axisline),*

*axis.ticks = element_line(size = axisticks),*

*axis.ticks.length = unit(0.5, "cm"))*

*# Residuals over time -----------------------------------------------------*

*residual_df <- GAM_final_data %>% dplyr::select(time, outcome_K_max_24, fit) %>%*

*mutate(residual = outcome_K_max_24-fit) %>% drop_na()*

*Figure3D <- ggplot(residual_df, aes(x = time, y=residual)) +*

*geom_point() +*

*ggtitle("3D") +*

*geom_hline(yintercept = 0, linetype='dashed') +*

*scale_x_continuous("Time (hours)", limits = c(-5,150), breaks = seq(0,144, 24), expand=c(0,0)) +*

*scale_y_continuous("Residuals ")+*

*theme_classic() +*

*theme(text = element_text(family = "serif"),*

*plot.title = element_text(face = "bold", size = titlesize),*

*axis.text.y = element_text(size = axistext, color = "black"),*

*axis.text.x = element_text(size = axistext, color = "black"),*

*axis.title.x = element_text(size = axistitletext, margin = margin(r = 13)),*

*axis.title.y = element_text(size = axistitletext, margin = margin(r = 10)),*

*axis.line = element_line(size = axisline),*

*axis.ticks = element_line(size = axisticks),*

*axis.ticks.length = unit(0.5, "cm"))*

*# Grid plot ---------------------------------------------------------------*

*tiff(here("Figure_3_grid.tiff"), width = 32, height = 32, units = "cm", res = 600, compression = "lzw")*

*print(grid.arrange(Figure3A, Figure3B, Figure3C, Figure3D, nrow= 2, ncol = 2, widths = c(1/2, 1/2)))*

*dev.off()*

References

1. Woo SA, Cragg A, Wickham ME, Villanyi D, Scheuermeyer F, Hau JP, Hohl CM. Preventable adverse drug events: Descriptive epidemiology. Br J Clin Pharmacol. 2020;86:291–302. doi:10.1111/bcp.14139.

2. Gates PJ, Meyerson SA, Baysari MT, Lehmann CU, Westbrook JI. Preventable Adverse Drug Events Among Inpatients: A Systematic Review. Pediatrics 2018. doi:10.1542/peds.2018-0805.

3. Jeon N, Staley B, Johns T, Lipori GP, Brumback B, Segal R, Winterstein AG. Identifying and characterizing preventable adverse drug events for prioritizing pharmacist intervention in hospitals. Am J Health Syst Pharm. 2017;74:1774–83. doi:10.2146/ajhp160387.

4. Haerdtlein A, Boehmer AM, Karsten Dafonte K, Rottenkolber M, Jaehde U, Dreischulte T. Prioritisation of Adverse Drug Events Leading to Hospital Admission and Occurring during Hospitalisation: A RAND Survey. J Clin Med 2022. doi:10.3390/jcm11154254.

5. Lusa L, Proust-Lima C, Schmidt CO, Lee KJ, Le Cessie S, Baillie M, et al. Initial data analysis for longitudinal studies to build a solid foundation for reproducible analysis. PLoS One. 2024;19:e0295726. doi:10.1371/journal.pone.0295726.

6. Welten M, Kroon MLA de, Renders CM, Steyerberg EW, Raat H, Twisk JWR, Heymans MW. Repeatedly measured predictors: a comparison of methods for prediction modeling. Diagn Progn Res. 2018;2:5. doi:10.1186/s41512-018-0024-7.

7. Meyer S. Developing and Applying a Design Framework to Prepare Electronic Health Record Data for Time-Series Modeling [Dissertation]. Michigan: The University of Michigan; 2021.

8. Steyerberg EW. Clinical Prediction Models. New York, NY: Springer New York; 2009.

9. Hunter RW, Bailey MA. Hyperkalemia: pathophysiology, risk factors and consequences. Nephrol Dial Transplant. 2019;34:iii2-iii11. doi:10.1093/ndt/gfz206.

10. Ben Salem C, Badreddine A, Fathallah N, Slim R, Hmouda H. Drug-induced hyperkalemia. Drug Saf. 2014;37:677–92. doi:10.1007/s40264-014-0196-1.

11. Reig-Lopez J, Tang W, Fernandez-Teruel C, Merino-Sanjuan M, Mangas-Sanjuan V, Boulton DW, Sharma P. Application of population physiologically based pharmacokinetic modelling to optimize target expression and clearance mechanisms of therapeutic monoclonal antibodies. Br J Clin Pharmacol. 2023;89:2691–702. doi:10.1111/bcp.15745.
